# Amoxicillin/Clavulanic Acid for the Treatment of Odontogenic Infections: A Randomised Study Comparing Efficacy and Tolerability versus Clindamycin

**DOI:** 10.1155/2015/472470

**Published:** 2015-08-02

**Authors:** Archiel Launch Tancawan, Maria Noemi Pato, Khamiza Zainol Abidin, A. S. Mohd Asari, Tran Xuan Thong, Puja Kochhar, Chandra Muganurmath, Monique Twynholm, Keith Barker

**Affiliations:** ^1^Tancawan Dental Clinic, Door 2 Cebu Coliseum Building, Leon Kilat Street, 6000 Cebu City, Philippines; ^2^East Avenue Medical Center, Dental Department, OPD Building, East Avenue, Diliman, 1100 Quezon City, Philippines; ^3^Klinik Pergigian Gunung Rapat, Unit Pakar Periodontik, Jalan Raja Dr. Nazrin Shah, 31350 Ipoh, Perak, Malaysia; ^4^Klinik Pergigian Cahaya Suria, Unit Periodontik, Tingkat 2, Bangunan Cahaya Suria, Jalan Tun Perak, 50050 Kuala Lumpur, Malaysia; ^5^People's Hospital 115, 527 Su Van Hanh Street, District 10, Ho Chi Minh City, Vietnam; ^6^GlaxoSmithKline Asia Pvt. Ltd., 252 Dr Annie Besant Road, Worli, Mumbai 400030, India; ^7^GlaxoSmithKline Pharmaceuticals Ltd., 5 Moore Drive, Research Triangle Park, NC 27709-3398, USA; ^8^GlaxoSmithKline Pharmaceuticals Ltd., Stockley Park West, 1-3 Iron Bridge Road, Heathrow, Uxbridge, Middlesex UB11 1BT, UK; ^9^GlaxoSmithKline Pharmaceuticals Ltd., GSK House, 980 Great West Road, Brentford, Middlesex TW8 9GS, UK

## Abstract

*Background*. Treatment of odontogenic infections includes surgical drainage and adjunctive antibiotics. This study was designed to generate efficacy and safety data to support twice daily dosing of amoxicillin/clavulanic acid compared to clindamycin in odontogenic infections. *Methods*. This was a phase IV, randomised, observer blind study; 472 subjects were randomised to receive amoxicillin/clavulanic acid (875 mg/125 mg BID, *n* = 235) or clindamycin (150 mg QID, *n* = 237) for 5 or 7 days based on clinical response. The primary endpoint was percentage of subjects achieving clinical success (composite measure of pain, swelling, fever, and additional antimicrobial therapy required) at the end of treatment. *Results*. The upper limit of two-sided 95% confidence interval for the treatment difference between the study arms (7.7%) was within protocol specified noninferiority margin of 10%, thus demonstrating noninferiority of amoxicillin/clavulanic acid to clindamycin. Secondary efficacy results showed a higher clinical success rate at Day 5 in the amoxicillin/clavulanic acid arm. Most adverse events (raised liver enzymes, diarrhoea, and headache) were similar across both arms and were of mild to moderate intensity. *Conclusion*. Amoxicillin/clavulanic acid was comparable to clindamycin in achieving clinical success (88.2% versus 89.7%) in acute odontogenic infections and the safety profile was consistent with the known side effects of both drugs. *Trial Registration*. This trial is registered with Clinicaltrials.gov identifier: NCT02141217.

## 1. Introduction

Odontogenic infections are one of the most prevalent diseases worldwide and the principal reason for seeking dental care. Dental prescriptions account for nearly 7% to 11% of all common antibiotic prescriptions [1]. The commonest emergency odontogenic infections are periapical abscess (25%), pericoronitis (11%), and periodontal abscess (7%) [2].

Odontogenic infections are mostly polymicrobial and frequently encountered odontopathogens are* Streptococci* spp.,* Corynebacterium* spp. and* Staphylococcus* spp.,* Prevotella* spp.,* Porphyromonas* spp.,* Fusobacterium* spp., and* Bacteroides* spp. [2, 3]. Therapeutic success in odontogenic infections is determined by the control of infection by surgical debridement and/or antimicrobial therapy which is indicated when there are clear signs of systemic involvement such as pyrexia or lymphadenopathy [2]. The polymicrobial nature of odontogenic infections necessitates the use of antibiotics active against both aerobic and anaerobic bacteria [4]. The antibiotics most commonly prescribed for acute dental abscesses are amoxicillin, penicillin, metronidazole, and erythromycin with clindamycin as an alternative in individuals allergic to the beta-lactam antibiotics [3].

Recently, published evidence suggests that penicillin and amoxicillin are being rendered increasingly less effective because of beta-lactamase producing bacteria. More than half of the Gram-negative anaerobic bacilli (including* Prevotella*,* Porphyromonas*,* Bacteroides*, and* Fusobacterium* spp.) are capable of producing beta-lactamase leading to treatment failures in dental infections [5]. Studies have revealed the presence of beta-lactamase producing species in 74–88% of patients with periodontitis [4]. Addition of a beta-lactamase inhibitor such as clavulanic acid to amoxicillin (Augmentin) confers resistance to beta-lactamases thereby extending the antibiotic spectrum to anaerobes such as* Prevotella* spp. and* Bacteroides* spp. anaerobes and to* Staphylococcus* spp. [4]. Efficacy of amoxicillin/clavulanic acid in the treatment of acute periapical abscess has been established in several studies [2, 4].

Clindamycin is a broad-spectrum antibiotic with activity against aerobic, anaerobic bacteria including coverage against beta-lactamase producing pathogens [6]. Clinical trials have demonstrated the efficacy of clindamycin in treating odontogenic infections [7–11]. Use of clindamycin in dental infections is based on careful patient selection in view of reported cases of pseudomembranous colitis (a rare but serious consequence of clindamycin).

Despite published evidence evaluating different oral formulations of amoxicillin/clavulanic acid in dental infections, there is limited published data on the use of twice daily dosing of 875/125 mg in odontogenic infections. Available evidence suggests that twice daily dosing with 875/125 mg amoxicillin/clavulanic acid results in a successful clinical outcome, better patient compliance, and less gastrointestinal upset, due to a reduction in the dose of clavulanic acid [1]. The aim of the current study was to assess the clinical efficacy and safety of amoxicillin/clavulanic acid 875/125 mg twice daily versus clindamycin 150 mg four times daily, for 5 or 7 days in dental infections.

## 2. Methods

### 2.1. Study Design

AUG117044 was a phase IV, randomised, parallel group, comparative, observer blind study to evaluate efficacy, safety, and tolerability of amoxicillin/clavulanic acid (875 mg/125 mg) and clindamycin (150 mg) in the treatment of acute odontogenic infection with or without abscess. The study was conducted in fifteen centres across four countries with four centres each in Malaysia, Philippines, and Vietnam and three in Thailand.

The study protocol, the informed consent, and other information that required preapproval were reviewed and approved by a national, regional, or investigational centre ethics committee or institutional review board, in accordance with the International Conference on Harmonisation of Technical Requirements for Registration of Pharmaceuticals for Human Use (ICH) Good Clinical Practice (GCP) and applicable country-specific requirements. Written informed consent was obtained from each subject prior to the performance of any study-specific procedures. The study was conducted in accordance with ICH GCP and all applicable subject privacy requirements and the ethical principles that are outlined in the Declaration of Helsinki 2008.

**Figure 1:**
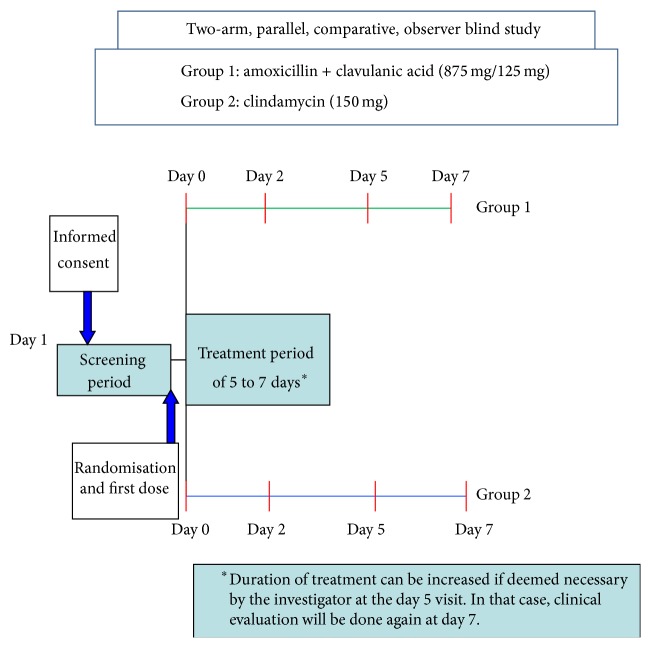
Study schema. Note: the study had a 1-day screening period (Day 1 to Day 0) during which eligibility was assessed and laboratory tests were performed. Randomisation occurred within 24 hours of screening at baseline (Day 0). Further visits (Day 2, Day 5, and Day 7) were calculated from the baseline/randomisation visit (Day 0).

A total of 472 subjects were randomised in a 1 : 1 ratio in each of the treatment arms. The study included a one-day screening period followed by a treatment period of five days that could be extended to seven days based on clinical response. Standard surgical intervention for odontogenic infection was permitted only before commencing study treatment. Eligible subjects were randomized on the day of their screening visit or within a day of screening. Efficacy and safety evaluations were performed on Day 2, Day 5, and/or Day 7 (based on treatment duration) (Figure 1). Study treatment included amoxicillin/clavulanic acid (875 mg/125 mg) administered twice daily or clindamycin (150 mg) administered four times daily along with meals for 5 or 7 days. Clinical efficacy (cure (cure was defined as complete resolution of signs and symptoms of infection present at baseline such that no additional antimicrobial therapy was required), improvement (improvement was defined as resolution of fever (if present at baseline) and >70% reduction in swelling and pain and improvement in other signs and symptoms such that no additional antimicrobial therapy was required), and failure (failure was defined as inability to improve the signs and symptoms of infection after seven days of therapy so that additional antimicrobial therapy was required)) of the study treatment was assessed based on the response shown by the subjects on the Visual Analogue Scale (VAS) scores of pain and swelling. Since these clinical efficacy parameters were based on symptomatic relief, an optimal study design would have been a double blind design. However, the different dosage regimens and formulations of the study drugs presented practical challenges for a double blind design. Based on these considerations, the study was designed to be an observer blind study with the investigator remaining blinded throughout the study period. An unblinded study team member was appointed for the study for drug dispensing and drug accountability and was also present during the clinic visits to ensure that the investigators remained blinded to treatment assignment. Adherence to the study design requirements was essential and no protocol waivers or exemptions were allowed during the study. This study did not require an independent data safety monitoring board and no interim analysis was performed.

### 2.2. Study Population

Inclusion criteria: the study enrolled subjects ≥18 years of age with a diagnosis of acute odontogenic infections (periapical abscess, acute periodontitis, and pericoronitis) that required antibiotic therapy. Radiographic evidence of odontogenic infection and dental pain on mastication were mandatory diagnostic criteria for enrollment. Exclusion criteria: subjects presenting with complicated odontogenic infections (such as osteomyelitis, dentocutaneous and dentoalveolar fistula, and facial-space swelling) or odontogenic infections secondary to traumatic injury or requiring hospitalisation, aggressive intravenous antimicrobial therapy, or local application of antimicrobials for the treatment of odontogenic infection were excluded. Further, patients with other key exclusion criteria such as conditions prone to infective endocarditis and those treated with systemic antibiotics within two weeks before the study of drug administration or injectable long acting antibiotics administered four weeks prior to study of drug administration were also excluded from study.

### 2.3. Study Variables

The primary objective of the study was the comparison of clinical efficacy of amoxicillin/clavulanic acid with clindamycin in subjects with acute odontogenic infection with or without abscess. This efficacy endpoint was based on the percentage of subjects achieving clinical success (cure or improvement) at the end of treatment (Day 5 or 7). The secondary endpoints of the study included percentage of subjects achieving clinical success at Day 5 and change in the VAS score for pain and swelling from baseline to Days 2, 5, and 7. Safety assessments included monitoring of adverse events (AEs) and serious adverse events (SAEs) from the start of study treatment until the end of study treatment. The antibiotic susceptibility of bacterial isolates obtained from pus specimens was recorded at baseline.

### 2.4. Statistical Analysis

A sample size of 205 evaluable subjects in each of the treatment arms provided 90% power to assess noninferiority for the primary endpoint. This was based on a noninferiority margin of 10%, assuming clinical success response rate in the comparator arm (clindamycin 150 mg) of 90% and a one-sided *α* = 2.5%. Considering a 15% drop-out rate, a final sample size of 236 subjects in each study group was chosen. Thus total randomised subjects in the study were 472 for a 1 : 1 treatment allocation in each study group to get at least 205 evaluable subjects in each arm.

The per-protocol (PP) population was a subset of Intent-to-Treat (ITT) population that had a postbaseline clinical success response assessment and did not report major protocol deviation(s). However, those subjects who discontinued from the study without any postbaseline assessment and where the reason for discontinuation was documented as “Lack of Efficacy” or “Treatment Failure” were included in the PP population with clinical success outcome treated as “Clinical Failure.” For noninferiority analysis, the PP population using observed case (OC) method was treated as the primary dataset. The assessment of noninferiority for clinical success response was based on two-sided 95% confidence interval (CI); the upper limit of two-sided 95% CI of the difference of proportion between the two treatments of less than 0.10 (10%) was set to conclude the noninferiority between the treatment arms. The analysis of the Intent-to-Treat-Efficacy (ITT-E) population (all randomised subjects with at least one postbaseline assessment of clinical success response) using the OC dataset was provided as a sensitivity analysis for the primary endpoint and was also used to evaluate secondary endpoints. The ITT population (all randomised subjects who received at least one dose of study medication) was the safety dataset for the study.

The investigator's judgment was considered decisive for the assessment of clinical improvement in a subject. In the event that the main signs and symptoms were cured or improved (complete resolution of fever and >70% reduction in swelling and pain) and there was “no change” or “worsening from baseline” in other signs and symptoms (such as increased leucocyte count/tooth mobility/lymphadenopathy), the investigator's opinion was sought as to whether additional antimicrobial therapy was required. Subjects that required no additional antimicrobial therapy per the investigators judgment were considered a “success” while those requiring additional antimicrobials were deemed as “failures.” The analysis based on the investigator's judgment of clinical success or failure was considered as the primary analysis for testing of noninferiority between the treatment groups and the analysis excluding the investigator's assessment for clinical success was presented as supportive analysis.

For sensitivity analysis, all subjects with an assessment of cure or improvement (complete resolution of fever, >70% reduction in swelling and pain) but with “no change” or “worsening from baseline” in other signs and symptoms (increased leucocyte count/tooth mobility/lymphadenopathy) were considered as “Clinical Failures” irrespective of the clinical judgment of investigator.

Clinical laboratory assessments were summarized on ITT population using OC approach for missing values. Liver function tests (LFTs) values were summarised after values were normalised. Any LFT parameter which was out of reference range for that particular laboratory was considered an adverse event (AE). However, AST, ALT, and alkaline phosphatase values >3 × upper limit of reference range (ULRR) and total bilirubin >1.5 × ULRR were considered to be of potential clinical concern (PCC) as defined by sponsor.

## 3. Results

### 3.1. Participants

A total of 510 subjects were screened for a planned enrollment of 472 subjects. Amongst the 472 randomised subjects, 235 (46.1%) subjects were randomised to the amoxicillin/clavulanic acid arm and 237 (46.5%) to the clindamycin arm. However, a total of 236 subjects received amoxicillin/clavulanic acid and 235 subjects received clindamycin (one subject randomised to the amoxicillin/clavulanic acid arm did not receive any study drug and two subjects randomised to the clindamycin arm incorrectly received amoxicillin/clavulanic acid). A similar proportion of enrolled subjects completed the study in both the treatment arms (223 (94.9%) in the amoxicillin/clavulanic acid arm and 229 (96.6%) in the clindamycin arm). A total of 11 (4.7%) subjects in the amoxicillin/clavulanic acid arm and 8 (3.4%) subjects in the clindamycin arm discontinued before study completion and the main reasons for discontinuations were as follows: AEs (one and two in amoxicillin/clavulanic acid and clindamycin arms, resp.), protocol noncompliance (one and four in amoxicillin/clavulanic acid and clindamycin arms, resp.), and meeting the withdrawal criteria (nine and two in amoxicillin/clavulanic acid and clindamycin arms, resp.).

### 3.2. Baseline Characteristics

Subjects recruited in the study were South East Asian in origin with similar age and sex distribution across both treatment arms. Periapical abscess was the predominant odontogenic infection across both arms (56.8% and 54.9% subjects in the amoxicillin/clavulanic acid and clindamycin arms, resp.). There was no significant difference in baseline characteristics such as pain, swelling, radiographic evidence of dental infection, and medical history/preexisting conditions between the treatment arms (Table 1).

### 3.3. Primary Efficacy Results

The primary efficacy analysis using the PP population demonstrated that the clinical efficacy of amoxicillin/clavulanic acid was noninferior to clindamycin, since the upper limit of two-sided 95% CI was within the protocol specified noninferiority margin of 10%.

The percentage of subjects achieving clinical success using the primary analysis population was 88.2% (95% CI: 83.0%, 92.2%) in the amoxicillin/clavulanic acid arm and 89.7% (95% CI: 84.6%, 93.5%) in the clindamycin arm. The treatment difference between the treatment arms was 1.5% (95% CI: −4.7%, 7.7%) using Miettinen and Nurminen method, 1.5% (95% CI: −4.9%, 8.0%) using Farrington and Manning method, and 1.5% (95% CI: −4.5%, 7.6%) using a two-sample proportion test. Since the upper limit of the two-sided 95% CI for between-group percentages differences was less than the prespecified noninferiority margin of 10%, noninferiority of amoxicillin/clavulanic acid to clindamycin with respect to clinical success was demonstrated. This result was also corroborated by sensitivity analysis using the ITT-E population (Table 2).

### 3.4. Secondary Efficacy Results

A slightly higher percentage of subjects achieved clinical success in the amoxicillin/clavulanic acid arm by Day 5 [76.8% (95% CI: 70.7%, 82.2%)] than the clindamycin arm [69.1% (95% CI 62.7%, 75.0%)] possibly indicating a faster response in subjects receiving amoxicillin/clavulanic acid arm as compared to subjects who received clindamycin.

The least square mean change in the VAS score for pain (using ITT-E dataset with OC approach) was maximum at Day 7 (6.38 and 6.34 in the amoxicillin/clavulanic acid and clindamycin arms, resp.) compared to Day 5 (5.49 and 5.38 in the treatment arms, resp.) and Day 2 (3.34 and 3.07, resp.) and it was similar between the treatment arms at each time point. Similar results were also noted for swelling (Table 3). A summary of VAS by visit and treatment arms demonstrated that higher mean percentage reduction in pain by Day 2 was achieved in the amoxicillin/clavulanic acid arm (49.5%) compared with (45.6%) the clindamycin arm. Similarly, a higher mean percentage reduction in swelling by Day 2 was achieved in the amoxicillin/clavulanic acid arm (43.6%) compared with the clindamycin arm (39.6%).

### 3.5. Microbiological Results

Pus specimens were obtained in 58 subjects who consented to microbiological sampling including two who were screen failures (25 in the amoxicillin/clavulanic acid arm and 31 in the clindamycin arm; the 2 screen failures were excluded). A total of 61 isolates were obtained from 56 samples, 26 in the amoxicillin/clavulanic acid arm and 35 in the clindamycin arm. Organisms isolated in both the treatment arms were similar and predominantly viridans streptococci group ((*n* = 24) including* Streptococcus oralis*,* Streptococcus mitis*, and* Streptococcus parasanguinis*),* Enterobacter* spp. (*n* = 10),* Klebsiella* spp. (*n* = 9),* Pseudomonas* spp. (*n* = 7), and* Staphylococcus* spp. (*n* = 5). CLSI breakpoints are not uniformly available for all isolates for both study drugs which posed a challenge in providing meaningful interpretation of susceptibility data.

### 3.6. Safety Results

**Figure 2:**
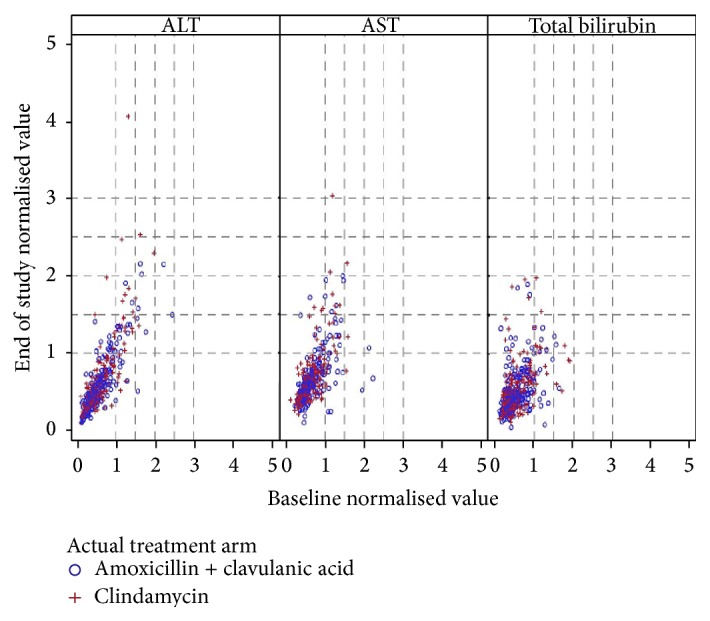
Scatter plot of LFT with respect to baseline and end of study by treatment. Normalised value = laboratory value/upper limit of normal reference range of the respective local laboratory.

A total of 243 treatment emergent AEs (TEAEs) were reported in 123 subjects in the amoxicillin/clavulanic acid arm and 236 events were reported in 124 subjects in the clindamycin arm. The most frequently observed TEAEs with frequency ≥3% were abdominal discomfort, raised liver enzymes (AST, ALT, and bilirubin), diarrhoea, dizziness, headache, increased appetite, and somnolence (Table 4). Generally the incidence of TEAEs was similar between treatment arms, except for diarrhoea and headache which were reported in slightly more patients in the clindamycin arm. Most AEs were of mild to moderate intensity. The incidence of drug related TEAEs in both the treatment arms was similar (165 AEs reported in 93 (39.4%) subjects and 171 AEs reported in 97 (41.3%) subjects in the amoxicillin/clavulanic acid and clindamycin arms, resp.). The most frequently reported drug related TEAEs were gastrointestinal disorders including abdominal discomfort, diarrhoea, nausea and vomiting, abnormal LFTs, increased appetite, somnolence, dizziness, and headache. Most of the related TEAEs were mild to moderate in intensity except for six events of severe intensity. These were elevated ALT, headache, and vomiting reported in the amoxicillin/clavulanic acid arm and burning sensation, hypertension, and hypersomnia in the clindamycin arm. All events had resolved by the end of the study except hypersomnia. A total of 89 subjects in the amoxicillin/clavulanic acid arm and 76 subjects in the clindamycin arm had AEs that remained ongoing at the end of the study. Increased LFT was the predominant ongoing AE and was present in 46 subjects in the amoxicillin/clavulanic acid arm and 40 subjects in clindamycin arm, respectively. The probable reason for the ongoing AEs could be the short duration of the study (7 to 8 days) and the lack of a planned follow-up visit after study treatment. No subjects in the study showed shift in ALT and AST from normal at baseline (with respect to local laboratory reference range) to PCC range (as defined in statistical analysis section) at end of the study whereas three subjects each in both the study arms showed shift in total bilirubin to PCC range at the end of the study. However, since these subjects only had increased bilirubin with no increase in ALT or AST, these were not of clinical concern. Four subjects in the clindamycin arm with high LFT parameter (one subject each for ALT and AST and two subjects for total bilirubin) at baseline remained in the PCC range at the end of the study (Figure 2). No events of SAE or death were reported in the study.

## 4. Discussion

The primary treatment in acute odontogenic infections is surgical drainage while antibiotics are an adjunct in patients showing signs of systemic involvement [12]. The polymicrobial component of odontogenic infection necessitates the use of antibiotics that are active against both aerobic and anaerobic bacteria and therefore are recommended [4]. The current study was aimed at comparing the clinical efficacy of amoxicillin/clavulanic acid (875 mg/125 mg) to clindamycin (150 mg) in subjects with acute odontogenic infections.

Amoxicillin/clavulanic acid (875 mg/125 mg) was administered twice daily for 5–7 days and was found to be noninferior or “comparable” to clindamycin (150 mg) administered four times daily. The overall clinical success seen with amoxicillin/clavulanic acid in the study (88.2% (95% CI: 83.0%, 92.2%)) was similar to results seen in other published studies. Success rates of 87% with amoxicillin/clavulanic acid 1 g twice daily and 96% with amoxicillin/clavulanic acid 625 mg thrice daily have been previously reported in other studies [13, 14]. A higher percentage of pain and swelling reduction (49.5% and 43.6%) was achieved in the amoxicillin/clavulanic acid arm compared to the clindamycin arm (45.6% and 39.6%) after two days of treatment. In a similar study aimed at demonstrating possible differences in the severity of symptoms after the use of amoxicillin and amoxicillin/clavulanic acid in dental ailments, pain was found to be less acute with amoxicillin/clavulanic acid [15]. Thus, these results indicate that amoxicillin/clavulanic acid given twice daily for the treatment of odontogenic infection serves as an appropriate treatment option with the potential advantage of an early clinical response.

Safety of the subjects, assessed throughout the study, showed that the overall incidences of TEAEs were similar across both the treatment arms. These were mainly events such as abdominal discomfort, diarrhoea, and raised liver enzymes. As per the available safety information of amoxicillin/clavulanic acid 875/125 mg, nausea and diarrhoea are commonly reported events [16]. Diarrhoea was seen in 8.1% of subjects in amoxicillin/clavulanic acid arm (compared to 11.9% in the clindamycin arm) and this is consistent with the known pharmacological effects of the drug and with the global prescribing information. Raised liver enzymes are a known but uncommon side effect (≥1/1,000 to <1/100) of amoxicillin/clavulanic acid. In the current study, a total of 34 subjects (14.4%) in the amoxicillin/clavulanic acid arm had raised liver enzymes posttreatment compared to 33 subjects (14.04%) in the clindamycin arm that were assessed by the investigator as related to the study drug. However, most of these events were of mild to moderate intensity and none of the subjects were considered to have any LFT values that were of clinical concern. Pseudomembranous colitis is a rare but serious side effect of both clindamycin and amoxicillin-clavulanic acid. However, literature evidence suggests that the incidence is particularly low when these antibiotics are given in outpatient care settings (6.7 cases/100,000 antibiotic exposures). In dental infections there is not much difference in the incidence of* C. difficile* colitis between the two drugs [6]. In the current study there were no cases of pseudomembranous colitis with either of the study drugs. There were no SAEs or deaths reported in this study.

One of the main limitations of the study was the use of an outcome measure based on a subjective score (VAS score) to assess pain and swelling to derive the composite clinical outcome. However, to overcome the subjectivity of the VAS score and to reflect real world practice, only subjects demonstrating >70% reduction in pain and swelling were considered for calculating clinical success response. Another limitation was not having a planned follow-up visit for the subjects after the end of the study visits. As a result, ongoing AEs typically key laboratory parameters such as liver enzymes could not be followed until resolution; however, most were mild and transient in nature.

## 5. Conclusions

Amoxicillin/clavulanic acid (875 mg/125 mg) administered twice daily was found to be comparable to clindamycin (150 mg) administered four times daily in achieving clinical success in acute odontogenic infections with or without abscess. It was also found to be well tolerated with a safety profile consistent with the known pharmacologic effects of amoxicillin/clavulanic acid and with that described in the global prescribing information.

## Figures and Tables

**Table 1:** Summary of demographics and baseline characteristics (ITT population).

Characteristic	Statistic	Randomised treatment arm	
		Amoxicillin/clavulanic acid (*N* = 234)	Clindamycin (*N* = 237)
Gender			
Male	*n* (%)	99 (42.3%)	94 (39.7%)
Female	*n* (%)	135 (57.7%)	143 (60.3%)
Geographic ancestry			
Asian: Central/South Asian heritage	*n* (%)	0	1 (0.4%)
Asian: East Asian heritage	*n* (%)	1 (0.4%)	4 (1.7%)
Asian: South East Asian heritage	*n* (%)	233 (99.6%)	232 (97.9%)
Age (years)	*N* (missing)	234 (0)	237 (0)
	Mean (SD)	33.1 (12.8)	32.6 (12.0)
	Median	29.2	28.9
	(Min, max)	(18.0, 74.8)	(18.1, 69.0)
Type of odontogenic infection			
Periapical abscess	*n* (%)	133 (56.8%)	130 (54.9%)
Acute periodontitis	*n* (%)	40 (17.1%)	37 (15.6%)
Pericoronitis	*n* (%)	62 (26.5%)	73 (30.8%)
Diagnostic criteria fulfilled			
Dental pain which is increased on mastication	*n* (%)	234 (100.0%)	237 (100.0%)
Swelling on alveolar mucosa	*n* (%)	226 (96.6%)	227 (95.8%)
Redness over involved region	*n* (%)	215 (91.9%)	219 (92.4%)
Increased tooth mobility	*n* (%)	82 (35.0%)	75 (31.6%)
Fever	*n* (%)	5 (2.1%)	5 (2.1%)
Malaise	*n* (%)	17 (7.3%)	21 (8.9%)
Cervical lymphadenopathy	*n* (%)	27 (11.5%)	26 (11.0%)
Elevated leucocyte count	*n* (%)	37 (15.8%)	37 (15.6%)
Baseline VAS score			
Dental pain	*N* (missing)	234 (0)	237 (0)
	Mean (SD)	6.5 (2.1)	6.5 (2.1)
	Median	7.0	6.5
	(Min, max)	(1.0, 10.0)	(0.5, 10.0)
Swelling	*N* (missing)	226 (8)	227 (10)
	Mean (SD)	4.2 (1.9)	4.5 (2.1)
	Median	4.0	4.0
	(Min, max)	(0.6, 10.0)	(0.1, 10.0)
Surgical intervention required prior to study treatment	*n* (%)	55 (23.5%)	55 (23.2%)

Percentage is calculated based on number of subjects in ITT population for each treatment arm and by actual randomised arm. As per randomisation, there were 234 subjects in amoxicillin/clavulanic acid arm and 237 in clindamycin arm but due to wrong randomisation process, two subjects were administered amoxicillin/clavulanic acid instead of clindamycin. Hence as per actual treatment received, there were 236 subjects in amoxicillin/clavulanic acid arm and 235 subjects in clindamycin arm.

**Table 2:** Primary efficacy endpoint: clinical success outcome at the end of the study.

Population	Clindamycin [% (95% CI)]	Amoxicillin/clavulanic acid [% (95% CI)]	Treatment difference		
			Miettinen and Nurminen method (primary) [% (95% CI)]	Farrington and Manning method [% (95% CI)]	Two-sample proportion test [% (95% CI)]
PP	89.7% (84.6%, 93.5%)	88.2% (83.0%, 92.2%)	1.5% ( 95 CI: −4.7%, 7.7%)	1.5% (−4.9%, 8.0%)	1.5% (−4.5%, 7.6%)

ITT-E	86.4% (81.3%, 90.5%)	85.5% (80.3%, 89.8%)	0.9% (−5.5%, 7.3%)	0.9% (−5.6%, 7.4%)	0.9% (−5.5%, 7.2%)

ITT (randomised treatment arm)	85.7% (80.5%, 89.9%)	83.3% (77.9%, 87.9%)	2.3% (−4.3%, 9.0%)	2.3% (−4.4%, 9.0%)	2.3% (−4.2%, 8.9%)

**Table 3:** Secondary endpoint: change in VAS scores for pain and swelling from baseline.

Secondary endpoint: change in VAS from baseline					
Assessment	Day *j*	Statistic	Amoxicillin/clavulanic acid (*N* = 228)	Clindamycin (*N* = 235)	Treatment difference
Change in pain at Day *j* from baseline		*N* (missing)	227 (1)	233 (2)	—
	Day 2	Least square mean	3.34	3.07	0.27
		Two-sided 95% CI (LCL, UCL)	(3.08, 3.61)	(2.81, 3.33)	(−0.10, 0.64)
		*N* (missing)	219 (9)	228 (7)	—
	Day 5	Least square mean	5.49	5.38	0.11
		Two-sided 95% CI (LCL, UCL)	(5.27, 5.71)	(5.16, 5.60)	(−0.20, 0.42)
		*N* (missing)	57 (0)	71 (0)	—
	Day 7^*∗*^	Least square mean	6.38	6.34	0.04
		Two-sided 95% CI (LCL, UCL)	(6.02, 6.74)	(6.02, 6.66)	(−0.44, 0.53)

Change in swelling at Day *j* from baseline		*N* (missing)	219 (2)	225 (1)	—
	Day 2	Least square mean	1.92	1.61	0.31
		Two-sided 95% CI (LCL, UCL)	(1.72, 2.11)	(1.42, 1.80)	(0.04, 0.57)
		*N* (missing)	214 (7)	223 (3)	—
	Day 5	Least square mean	3.68	3.60	0.08
		Two-sided 95% CI (LCL, UCL)	(3.51, 3.85)	(3.43, 3.76)	(−0.16, 0.32)
		*N* (missing)	55 (0)	68 (0)	—
	Day 7^*∗*^	Least square mean	4.21	4.61	−0.39
		Two-sided 95% CI (LCL, UCL)	(3.94, 4.49)	(4.36, 4.86)	(−0.77, −0.02)

Note: change from baseline in VAS for assessment of pain and swelling was analysed using a mixed model for repeated measures (MMRM) with restricted maximum likelihood and an unstructured covariance matrix. The baseline VAS score was used as a covariate. Treatment groups and nominal days (visits) were considered as fixed effects and interaction effect was considered between treatment and visit (days). Data for “Day 7” is summarised for subjects who continued the study up to Day 7. OC method was used for missing values where the missing value was kept as missing except for early withdrawal.

“*j*” indicates Day 2, or Day 5, or Day 7 as applicable.

LCL: lower confidence limit, UCL: upper confidence limit.

*∗* means the interaction effect between treatment and visits (days).

**Table 4:** Summary of commonly observed treatment emergent AEs (≥3% in either of the arms).

MedDRA preferred term	Amoxicillin/clavulanic acid (*N* = 236)	Clindamycin (*N* = 235)
Total number of treatment emergent AEs	243	236
Subjects who experienced at least one AE	123 (52.1%)	124 (52.8%)
Abdominal discomfort	11 (4.7%)	7 (3.0%)
Alanine aminotransferase increased	26 (11.0%)	24 (10.2%)
Aspartate aminotransferase increased	24 (10.2%)	20 (8.5%)
Blood bilirubin increased	12 (5.1%)	13 (5.5%)
Diarrhoea	19 (8.1%)	28 (11.9%)
Dizziness	18 (7.6%)	14 (6.0%)
Headache	8 (3.4%)	14 (6.0%)
Increased appetite	20 (8.5%)	15 (6.4%)
Nausea	7 (3.0%)	4 (1.7%)
Somnolence	19 (8.1%)	17 (7.2%)
